# Stem Cell Aging in Skeletal Muscle Regeneration and Disease

**DOI:** 10.3390/ijms21051830

**Published:** 2020-03-06

**Authors:** Hiroyuki Yamakawa, Dai Kusumoto, Hisayuki Hashimoto, Shinsuke Yuasa

**Affiliations:** 1 Department of Cardiology Keio University School of Medicine, Tokyo 160-8582, Japan; yamakawa@cpnet.med.keio.ac.jp (H.Y.); d-kusumoto@nifty.com (D.K.); hisayuki.hashimoto@gmail.com (H.H.); 2 Center for Preventive Medicine, Keio University School of Medicine, Tokyo 160-8582 Japan

**Keywords:** skeletal muscle, regeneration, MuSC, stem cell, aging

## Abstract

Skeletal muscle comprises 30–40% of the weight of a healthy human body and is required for voluntary movements in humans. Mature skeletal muscle is formed by multinuclear cells, which are called myofibers. Formation of myofibers depends on the proliferation, differentiation, and fusion of muscle progenitor cells during development and after injury. Muscle progenitor cells are derived from muscle satellite (stem) cells (MuSCs), which reside on the surface of the myofiber but beneath the basement membrane. MuSCs play a central role in postnatal maintenance, growth, repair, and regeneration of skeletal muscle. In sedentary adult muscle, MuSCs are mitotically quiescent, but are promptly activated in response to muscle injury. Physiological and chronological aging induces MuSC aging, leading to an impaired regenerative capability. Importantly, in pathological situations, repetitive muscle injury induces early impairment of MuSCs due to stem cell aging and leads to early impairment of regeneration ability. In this review, we discuss (1) the role of MuSCs in muscle regeneration, (2) stem cell aging under physiological and pathological conditions, and (3) prospects related to clinical applications of controlling MuSCs.

## 1. Introduction

Skeletal muscle is one of the largest organs in the human body, and the weight of skeletal muscle is approximately 30–40% of the human body [1]. Skeletal muscle plays a critical role in voluntary movement and has several other functions such as metabolic and endocrine functions. Healthy skeletal muscle is crucial for human life. Skeletal muscle increases in cell number and cellular size during development. In the adult stage, skeletal muscle maintains its function and size through regeneration after muscle injury. Even after severe and repetitive muscle injuries, appropriate regeneration can recover muscle function. Therefore, the imbalance between muscle injury and regeneration causes deterioration of muscle function, resulting in the development of multiple diseases.

Skeletal muscle is composed of bundles of multinucleated muscle fibers. Each myofiber is formed by the fusion of mononucleated myoblasts. Adult skeletal muscle has its own stem cell population, namely muscle satellite (stem) cells (MuSCs). Under sedentary conditions in the adult stage, MuSCs are mitotically quiescent and reside beneath the basal lamina of the myofiber [2]; this position between the myofiber and the surrounding extracellular matrix is crucial for maintaining the stem cell state [3]. After muscle injury, quiescent MuSCs promptly get activated, resulting in proliferation and their differentiation into myoblasts. Hence, myoblast fusion is critical not only for skeletal muscle development but also for regeneration [4].

Quiescent MuSCs differentiate into mature myofibers in a stepwise fashion with serial expression of myogenic transcription factors [5,6]. This differentiation program resembles the process of embryonic skeletal muscle development [5,6]. Certain rare populations of quiescent MuSCs and several types of progenies can be identified by unique expression patterns of several marker genes in combination [7]. These genes are now used as markers to identify the cell status, and they also play roles in biological function, such as specific gene expression, proliferation, differentiation, migration, and metabolism [8].

Thus, MuSCs play a critical role in muscle regeneration. However, the function of MuSCs gradually declines during physiological and pathological aging. Although loss of the muscular regenerative capacity in aging is partly due to this impairment of MuSC function, the precise mechanism of how stem cell function is maintained and impaired remains unclear [9]. Here, we discuss the role of MuSCs in muscle regeneration, stem cell aging under physiological and pathological conditions, and prospects related to clinical applications of controlling MuSCs.

## 2. MuSCs

### 2.1. MuSC Quiescence and Differentiation

Adult MuSCs are a rare population of cells that resides in a quiescent state. However, after injury, MuSCs get rapidly activated whereby proliferation and differentiation are induced, resulting in recovery of the damaged tissue. Although there are several studies reporting that other types of stem cells contribute to muscle regeneration, such as bone marrow stem cells, or mesenchymal stem cells, MuSCs play a pivotal role in the homeostasis of adult skeletal muscle. For example, MuSC depletion in adult mice induces severe muscle damage with reduced regeneration potential [10].

**Figure 1:**
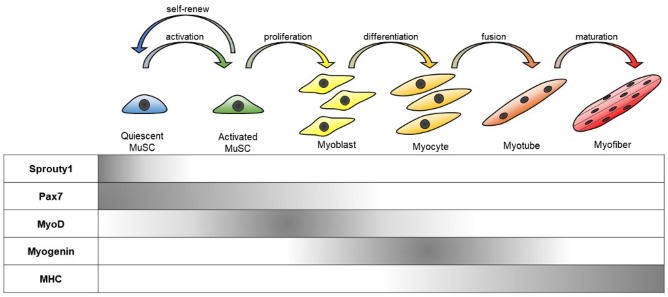
Stepwise muscle differentiation from muscle satellite (stem) cells (MuSCs). Quiescent MuSCs express Pax7 without expression of MRFs. Activated MuSCs proliferate and irreversibly differentiate into proliferating myoblasts that express the myogenic transcription factors including MyoD. Myoblasts further differentiate into myocytes with the expression of other MRFs such as Myogenin and MRF4. Then, myoblasts cease proliferation and fuse to form a multinucleated myotube. Myotubes undergo further maturation and bundle together as myofibers.

MuSCs are heterogeneous in terms of their developmental origin, functional diversity, and differentiation status [11]. Nevertheless, MuSCs can be characterized by a combination of several genetic markers, including Paired box protein, *Pax7* (regarded as a definitive MuSC marker), and muscle regulatory factors (MRFs: MyoD, Myf5, Myogenin, and MRF4) [8,12]. Pax7 is a critical regulator of MuSC survival and is ubiquitously expressed in all states of MuSCs. For instance, quiescent MuSCs express Pax7 but lack the expression of other myogenic markers including MyoD, a key transcription factor for myogenesis. Meanwhile, MuSCs in the post injury state express both Pax7 and MyoD. Activated MuSCs can further differentiate into myogenic progenitors that express MRFs, namely myoblasts, or turn back into the quiescent state in association with a loss of MyoD expression. When Pax7 expression declines in these myogenic progenitors, they begin to differentiate into myocytes and their fusions generate new multi-nucleated myofibers [5] (Figure 1).

Quiescent MuSCs govern the homeostasis of skeletal muscle cells and they are essential for maintaining MuSCs throughout life. For example, after injury, part of the activated MuSCs revert to the quiescent state [13,14] in order to maintain the balance of the MuSC population. So how is this balance regulated? There are several mechanisms investigated to understand the system of MuSC maintenance. Sprouty1 (Spry1), a receptor tyrosine kinase (RTK) signaling inhibitor, is exclusively expressed in quiescent MuSCs. RTK is a receptor for growth factors, cytokines, and hormones. RTK signaling plays a critical role for cellular proliferation, migration, differentiation, survival, and death in many tissues. There are many RTK ligands that are potent activators of MuSCs. The expression of *Spry1* is downregulated in activated MuSCs and upregulated in reverted quiescent MuSCs. The disruption of *Spry1* in adult MuSCs prevents their reversion back to the quiescent state and results in a reduction of the MuSC pool to homeostatic levels after muscle injury [15].

The Notch signaling pathway is a highly conserved cell transduction pathway amongst species and plays an important role in various cellular functions [16]. Notch receptors are transmembrane proteins comprised of an extracellular domain and an intracellular domain (NICD). Notch signaling is activated when Notch ligands bind to Notch receptors, leading to cleavage of the NICD and its translocation into the nucleus where it acts as coactivators of transcription factors. In the adult stage, MuSCs express the Notch receptors, *Notch1*, *Notch2*, and *Notch3*. Genetic disruption of each Notch receptor in mice show embryonic lethality or developmental defects in multiple organs including skeletal muscle, which explains the importance of Notch signaling in skeletal muscle development [17,18]. Additionally, the Notch signaling pathway maintains quiescence and suppresses proliferation in MuSCs. Mutations in genes related to Notch signaling affect the subpopulation of MuSCs, which suggests that Notch signaling is essential for the homeostasis of MuSCs [19]. Disruptions in Notch signaling enable MuSCs to escape quiescence and express markers of proliferation and differentiation in the absence of injury or stimulation [20]. The satellite cell-specific depletion of recombination signal binding protein for immunoglobulin kappa J region (RBPJ), the DNA binding factor essential for mediating canonical Notch signaling, leads to spontaneous differentiation and progressive loss of satellite cells [20,21]. Notch activation antagonizes myogenesis and maintains MuSC quiescence by inducing expression of extracellular components within the environment [22,23]. The transcription factor, Forkhead box protein O3 (FoxO3), is expressed in quiescent MuSCs and supports the quiescent state by activating Notch signaling [24]. Nevertheless, the maintenance of MuSC quiescence requires other signaling, epigenetic, transcriptional, and post-translational regulators.

### 2.2. Cellular Interactions in the Maintenance of MuSCs

In addition to MuSCs, other cell types, such as fibro-adipogenic precursors (FAPs), endothelial cells (ECs), fibroblasts, pericytes, and several types of immune cells (neutrophils, M1 macrophages, eosinophils, M2 macrophages, regulatory T cells (Tregs)) play important regulatory roles during skeletal muscle regeneration [25]. For instance, the state of MuSCs is regulated by their surrounding niche, which includes extracellular matrix and multiple cell types [26]. These cells regulate MuSC status, such as activation, proliferation, and differentiation after muscle injury [27]. Conversely, neighboring cells surrounding the activated MuSCs can also act on MuSCs to promote their quiescence [28]. The activation of MuSCs is triggered by several factors released by resident cells and infiltrating inflammatory cells in response to muscle injury [29,30,31]. These environmental signals induce rapid expression of MRFs that control the transcriptional programs of activated MuSCs, such as cell-cycle progression, metabolic processes, and responses to the immune system [31,32,33,34].

Several types of immune cells rapidly accumulate in injured sites in response to the factors released from degenerated muscles [35]. Neutrophils are the first inflammatory cells to invade the injured muscle [36]. After neutrophil infiltration, macrophages become the next predominant inflammatory cells present, and the main role of macrophages is to engulf cellular debris. Infiltrated macrophages also secrete several cytokines that stimulate MuSC proliferation [36,37]. Muscle damage rapidly recruits eosinophils, which secrete IL-4 to indirectly activate MuSCs via FAPs [38]. Tregs accumulate to injury sites and play a role in muscle regeneration. Depletion of Tregs during muscle regeneration prolongs the proinflammatory process and impairs muscle repair, while increased Treg activities improve muscle regeneration through the expression of Amphiregulin, which acts on MuSCs [39].

FAPs are not commonly characterized cell types in other tissues. FAPs are characterized as muscle-resident non-myogenic progenitors of mesenchymal origin that are marked by expression of platelet-derived growth factor receptor alpha (PDGFRα) and stem cell antigen-1 (Sca1) [40,41]. FAPs are quiescent in intact muscles but rapidly proliferate after injury in adjacent regenerative myofibers [40]. FAPs are the chief mediators of fat and fibrotic tissue accumulation in skeletal muscle in pathologic conditions [42]. FAPs stimulate MuSCs and enhance myogenic differentiation through pro-differentiation signals, IGF-1, interleukin-6 (IL-6), Wnt1, Wnt3a, and Wnt5a [43]. Although some reports exist, the myogenic potential of FAPs or FAP-like cells [44,45] still remains controversial and further studies are needed.

Myogenesis and angiogenesis are coupled by interacting ECs and myogenic progenitors during skeletal muscle regeneration [46]. The necessity of ECs in the homeostasis of MuSCs is unclear, but accumulating data suggest that ECs have a direct role in affecting MuSC status. Most MuSCs remain in proximity to capillaries regardless of their state of quiescence, proliferation, and differentiation [47]. During muscle regeneration, MuSCs recruit capillary ECs and vascular ECs gradually increase after injury. ECs support the regenerative process of MuSCs through paracrine effects and direct interactions. Several secreting factors from ECs are involved in the regenerative process, including angiopoietin-1 (Ang-1), insulin-like growth factor-1 (IGF-1), hepatocyte growth factor (HGF), and vascular endothelial growth factor (VEGF) [27,28,47,48,49]. Membrane type Notch ligand, Dll4, is expressed in ECs after injury and has a critical role in MuSCs. ECs are important by their direct interactions with MuSC self-renewal and differentiation [50].

After injury, fibroblasts rapidly proliferate in the proximity of MuSCs. These fibroblasts contribute to MuSC differentiation by promoting the fusion of myoblasts [51]. Ablation of MuSCs impairs muscle regeneration and leads to dysregulation of fibroblasts resulting in increased fibrosis. Ablation of fibroblasts leads to premature MuSC differentiation, depletion of the early pool of MuSCs, and smaller regenerated myofibers [52]. Thus, fibroblasts support MuSCs in many aspects, but it remains unclear how fibroblasts support the homeostasis of MuSCs.

Pericytes are mural cells surrounding blood vessels, adjacent to endothelial cells. Pericytes play critical roles in maturation and maintenance of vascular branching morphogenesis [53]. Most capillaries are associated with pericytes in adult muscle. During postnatal growth, newly formed vessels with pericytes associate with MuSCs, as myofibers increase in size and MuSCs enter into quiescence. Pericytes promote myogenic differentiation through IGF-1 and quiescence through Ang-1. Ablation of muscle pericytes in mice lead to myofiber hypotrophy and to impaired establishment of stem cell quiescence [54]. Additionally, degenerative and regenerative processes in muscles are related to an increase in the pericyte population in humans [55]. Data indicate that pericytes also play a critical role in skeletal muscle regeneration.

**Figure 2:**
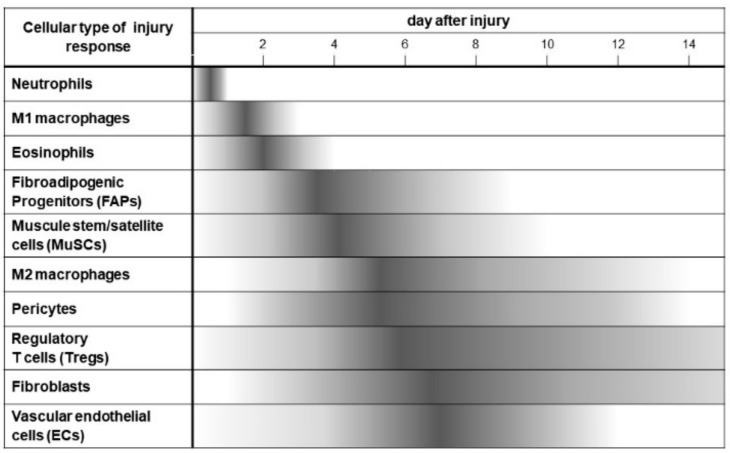
The cellular dynamics during muscle regeneration after injury.

In summary, Figure 2 shows the cascade of cellular dynamics that occurs from day 0 to 14 after skeletal muscle injury. One of the main goals of the cellular cascade is to nurture MuSCs to activate, expand, differentiate, maturate, and return to quiescence. Cells in this regenerative environment have coordinated responses to secrete multiple cytokines that target MuSCs, express membrane proteins that interact with MuSCs, and express extracellular matrix that create the niche to fulfill this regenerative endeavor and rebuild functional skeletal muscle [27].

Figure 2 represents the cellular dynamics in the injured muscle during the first 14 days after muscle injury. In the regenerative response, many types of cells serially infiltrate into injured muscle, such as neutrophils, M1 macrophages, eosinophils, FAPs (fibro-adipogenic progenitors), MuSCs (muscle stem cells), M2, macrophages, pericytes, regulatory T cells (Tregs), fibroblasts, and vascular endothelial cells (ECs). In an orderly fashion, these processes regulate debris removal, inflammation, and regeneration by direct cellular action and cellular interaction.

## 3. Aging in MuSCs

### 3.1. The Decline of Regenerative Capacity with Aging

Skeletal muscle has an outstanding regenerative capacity that relies on MuSCs, but this regenerative capacity after injury declines with aging [56,57,58]. This is due to an age-associated loss of function of MuSCs. Age-related changes within the skeletal muscle tissue and the host environment, such as an increased prevalence of inflammation, also affect MuSC function in response to injury [57]. The ability of MuSCs to become activated and proliferate after injury has a regenerative correlation to aging [59,60]. Aged MuSCs are also more prone to undergo senescence or apoptosis than young MuSCs [61]. In terms of the ability of MuSCs to differentiate, the adipogenic differentiation program is enhanced in cultured, aged MuSCs [62,63]. In the context of acute injury, symmetric and asymmetric cell division promote the expansion of MuSCs and maintain homeostasis of the stem cell compartment [64,65,66]. Impairment of this process in aged muscle leads to an impaired propensity to proliferate and produce myoblasts necessary for muscle regeneration [67]. While there are reports demonstrating decreases in the number of MuSCs during aging [68,69,70,71], conflicting reports show no significant differences in the number of MuSCs between young and aged mice [72,73,74]. Additionally, since MuSCs are very rare and the number of MuSCs differs in the type and location of skeletal muscles, it is difficult to reach conclusions on the frequencies of MuSCs within young and aged mice.

The decline of MuSC regenerative capacity is due to age-associated extrinsic/environmental changes as well as cell-intrinsic/autonomous changes [67,75,76]. As extrinsic factors, inflammatory responses, extracellular components, and changes in interacting cell types definitely affect the function in MuSCs. MuSC function is also impaired by cell-intrinsic damages including oxidative stress, DNA damage, modified signaling pathways, damage to proteins, and altered metabolism [77]. An accumulation of cell intrinsic damages leads to a “point of no return” in aged MuSCs as they go into a pre-senescent state or they undergo apoptosis. Alterations in several intracellular signaling pathways in aged MuSCs affect their self-renewability [66,78,79,80]. The functional decline of MuSCs is partly due to the activation of FGF2 [81], TGF-b- [75], WNT-pathways [76], JAK/STAT3 [79], p16^INK4a^ [77,82], and p38 [66,83]. Those pathway modulations could be a therapeutic target for muscle regenerative therapy in elderly. These cell autonomous and non-cell autonomous changes in aged MuSCs underlie the abnormal regeneration of aged skeletal muscle.

### 3.2. Sarcopenia and MuSCs

Skeletal muscle aging is characterized by a loss of volume and function, which is referred as “sarcopenia”. Many factors directly or indirectly affect the muscle aging phenotype, and contribute to sarcopenia, such as nutritional, hormonal, metabolic, neurological, and immunological alterations [84]. Sarcopenic muscles show reduced numbers of myofibers and hypotrophic myofibers, infiltration with adipose and, at later stages, fibrotic tissue [85]. Some groups have reported the contribution of MuSCs in sarcopenia, but it remains controversial [86,87]. A decline in MuSC function and/or number during aging leads to loss of nuclei in large fibers [88]. Experimental loss of MuSCs does not accelerate sarcopenia in mice, but increases age-dependent muscle fibrosis [86]. MuSCs lose their regenerative potential with age [89], and this is particularly pronounced in sarcopenic muscle [90]. At advanced geriatric age, the function of MuSCs sharply declines and sarcopenia becomes prominent [91]. These data suggest that although MuSCs may not play a crucial role in sarcopenia, the existence of MuSCs in a healthy state is necessary for the maintenance of a healthy muscle.

There are many similarities and differences between human and mouse in skeletal muscle homeostasis and pathogenesis [92]. MuSCs obtained from young and elderly people showed similar potential for proliferation and differentiation [93,94,95]. Aged human skeletal muscle showed a decline in the number of MuSCs [85], but aged human MuSCs are susceptible to apoptosis [96]. Whole-genome sequencing of human MuSCs revealed that mutations increased with aging and would be a driving force in the aging phenotype of skeletal muscle [97].

### 3.3. Stem Cell Aging in Diseased Condition

Many diseases such as cancer, congestive heart failure, chronic obstructive pulmonary disease, renal failure, chronic infectious diseases, neuromuscular diseases, chronic inflammatory diseases, and acute critical illness, induce skeletal muscle wasting (i.e., atrophy). Disease-induced muscle atrophy (cachexia) is associated with increased morbidity and mortality and a decreased quality of life [98]. Those diseases have multi-faceted mechanisms on muscle atrophy such as inflammation, oxidative stress, metabolic change, insufficient/unbalanced nutrition, and immobility, which definitely affect MuSC function. A comprehensive strategy is necessary to treat disease-induced muscle atrophy.

Mechanical unloading of muscle causes disuse atrophy and leads to reduced muscle mass without fiber attrition [99]. MuSCs and myonuclei are integrally involved in skeletal muscle responses to environmental changes by mechanical unloading. There are several reports about the number of MuSCs in disuse atrophic muscle, but results remain inconclusive [100,101,102]. The number of MuSCs is varied during mechanical unloading [103]. MuSCs taken from atrophied muscle cells showed reduced proliferation and differentiation ability into normal myonucleated myotubes in vitro [104]. In disused atrophied muscle, regenerative potential in response to muscle injury was also impaired enough to recover normal muscle size [105].

Adult skeletal muscle has full regenerative potential after acute injury at a young age [106]. Duchenne muscular dystrophy (DMD) is one of the most commonly inherited muscle diseases in humans [107,108]. DMD is caused by mutations in the gene encoding dystrophin, which links the internal muscle cytoskeleton to the extracellular matrix, enabling lateral transmission of force from within muscle cells to the surrounding matrix [109]. Mutations in the dystrophin lead to dystrophin deficiency at the myofiber membrane, where muscle fibers progressively degenerate and become fragile to mechanical stress [108]. In early stages of the diseases, healthy MuSCs regenerate the damaged skeletal muscle in response to muscle fiber degeneration. Repetitive muscle injury induces a degeneration–regeneration cycle that leads to early aging in MuSCs [110,111]. As the disease progresses, MuSCs gradually lose their replicative capacity, which is limited by telomere shortening so that at later stages of the disease there is a decrease in the number of MuSCs as well as a decrease in the potential replicative capacity accompanied by a hostile fibrotic environment [112]. In the late stage of DMD, muscle regeneration cannot compensate for the loss of skeletal muscle. However, the number of MuSCs after injury in DMD patients is comparable to aged-matched healthy individuals [113,114]. These data suggest that the quantity as well as the quality of MuSCs is important for proper muscle regeneration in DMD patients. In order to develop an effective therapy for DMD, it is also important to understand the molecular mechanism of DMD pathology. Dystrophin is highly expressed in activated MuSCs, where it associates with the serine–threonine kinase, Mark2, an important regulator of cell polarity [115]. The polarity in MuSCs is important for asymmetric cell division and MuSC self-renewal [116,117]. Dystrophin deficiency induces dysregulation of p38/Carm1 localization in MuSCs, resulting in modified epigenetic gene regulation [65]. In DMD, establishment of polarity in activated MuSCs is disrupted, resulting in the abrogation of asymmetric cell divisions, mitotic abnormalities, and inefficient generation of myogenic progenitors [115,118].

For DMD as well as other neuromuscular diseases, we are now stepping into a new era where several pharmacological and gene therapy trials are ongoing. In DMD, several mutations in *Dystrophin* lead to the loss of Dystrophin protein. Gene therapy to reinstate functional Dystrophin expression has become feasible with the development of adeno-associated vectors (AAVs) to deliver short forms of the *Dystrophin* gene, namely micro-dystrophins. [119]. AAVs are also used to systemically deliver CRISPR-Cas9 to specifically correct mutations in *Dystrophin* [120]. DMD is often caused by frameshift mutations causing premature termination in *Dystrophin*. In these cases, several chemical drugs are used for the exon skipping approach to express functional short form of Dystrophin [121]. The CRISPR-Cas9-mediated gene delivery system is also applied to exon skipping/deletion approaches [122]. Theoretically, these approaches are promising but it is important to treat properly MuSCs to preserve its effect for a long term in whole body.

## 4. Prospects for Clinical Application Utilizing MuSCs

### 4.1. Cell-Autonomous Rejuvenation Strategy

**Figure 3:**
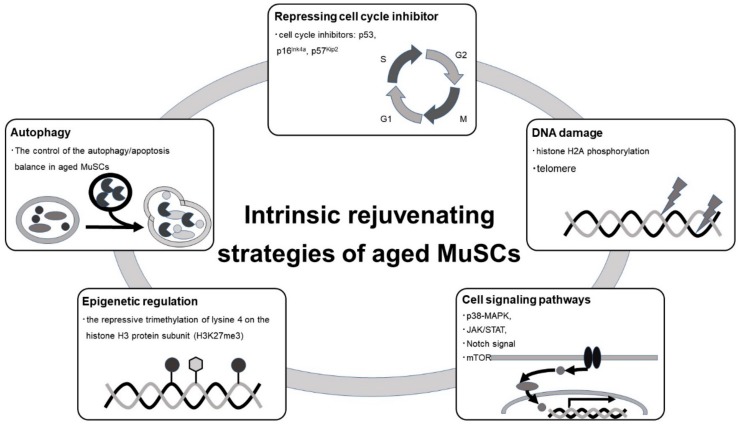
Intrinsic rejuvenating strategies of aged MuSC. MuSCs are controlled by intrinsic effectors including cell cycle regulator, autophagy, epigenetic regulation, cell signaling pathways, and DNA damage. Aged MuSCs would regain the capacity for self-renewal, and proliferation and differentiation by regulating these intrinsic effectors.

Intracellular changes in MuSCs that correlate with aging include changes in the transcriptome, epigenome, post translational modification, and signal transduction. These changes induce cell-autonomous aging and are potential targets for rejuvenation in aged MuSCs. Figure 3 shows major strategies targeting intracellular processes for rejuvenation in aged MuSCs [123].

Increased expression of several cell cycle inhibitors, such as p53 [124,125], p16^Ink4^ [77], and p57^Kip2^ [126] leads to an irreversible cell cycle arrest and promotes cellular senescence. p53 levels also regulate the balance between differentiation, proliferation, and quiescence in MuSCs [127]. Controlling p53 expression improves the proliferative capacity of MuSCs in certain diseases [124]. Additionally, expression of p16^Ink4^ accumulates with age, and suppression of p16^Ink4^ improves the function of aged stem cells and prevents cellular senescence [77]. Expression of p57^Kip2^ is directly activated by MRFs in myoblasts through muscle-specific regulatory element [18,126]

Autophagy is an evolutionarily conserved catabolic function that plays a role in cellular homeostasis by lysosomal degradation and recycling of intracellular macromolecules and organelles [128]. Autophagy is activated in MuSCs during the early, compensatory regenerative stages of DMD. A progressive reduction of autophagy was observed during DMD disease progression, in conjunction with a functional disturbance of MuSC-mediated regeneration and accumulation of fibrosis [129]. Autophagy is critical for the activation and proliferation of MuSCs, acting as a temporary energy source [130]. Lack of autophagy in physiologically aged MuSCs or genetic impairment of autophagy in young MuSCs causes senescence [131]. Hence, the control of the autophagy/apoptosis balance in aged MuSCs rescues muscle regeneration potential [132].

The epigenome also plays a central role in cellular function. Epigenetic regulation, including DNA and histone modification, controls global gene expression patterns in cells. Alterations in the epigenome that occur with aging can impinge on cellular processes in aged organisms [133,134]. Global chromatin modification patterns are different between young and aged MuSCs, and these age-dependent epigenomic changes lead to a functional decline in MuSCs [32,135].The epigenetic response to stress also differs between young and aged MuSCs, so targeting these pathways may rescue the aged phenotype in MuSCs [136]. In fact, epigenome-specific enzyme blockers could be useful for treating stem cell aging in MuSCs [137,138,139].

Intracellular signals directly influence all aspects of cellular function, including stem cell functions such as quiescence, proliferation, and differentiation. Signal transduction pathways involving p38-MAPK [66], Janus kinase (JAK)/signal transducers and activators of transcription (STAT) [79,80], Notch, and mechanistic target of rapamycin kinase (mTOR) [140], control stem cell function. These signaling pathways are activated or inactivated with age, which lead to aging phenotypes in MuSCs [141]. Certain stresses to skeletal muscle induce the release of cytokines, hormones, and growth factors that activate intracellular signal transduction pathways in MuSCs. Increased activation of STAT3 has been identified in muscle wasting conditions [80]. The JAK/STAT pathway is activated in aged MuSCs and its inhibition restores MuSC function [79,142]. p38α/βMAPK signaling is involved in cell self-renewal of MuSCs after injury, and MuSCs from aged mice exhibit activation of p38α/β MAPK signaling [83,116]. Pharmacological inhibition of p38α/βMAPK ameliorates age-associated self-renewal defects [66]. Notch signaling is deactivated in MuSCs during aging due to the suppression of Delta expression [67,143]. Delta/Notch signaling suppression leads to decreased activity of MuSCs and impairment in muscle regeneration [140]. Stimulation of Notch signaling restores the proliferative and regenerative ability of MuSCs [140].

DNA damage markers, such as histone H2A phosphorylation and comet tails, significantly increase in MuSCs during aging [143,144]. DNA damage induces apoptosis in MuSCs [145]. Pharmacologic activation of p53 suppresses DNA damage, ameliorates cell death, and limits the expansion of MuSCs in aged mice [143]. Accumulation of DNA damage in aged stem cells reduces their regenerative potential and could be a therapeutic target in aging MuSCs.

### 4.2. Non-Cell Autonomous Rejuvenation Strategy

Somatic stem cells including MuSCs reside in the niches and their cellular characteristics are directly regulated by niche [146,147,148]. The niche components are affected in age dependent manner and could be a target to rejuvenate aged MuSCs. For instance, aged MuSC niches expresses fibroblast growth factor 2 (Fgf2) and drives MuSCs to escape quiescence and lose their self-renewing capacity. Inhibition of FGF signaling rescues the aging phenotype, self-renewal capacity in aged MuSCs [81]. Aged MuSC niches also have reduced expression of fibronectin, an extracellular matrix protein. Thus, fibronectin-mediated signaling is impaired in aged MuSCs, leading to detrimental consequences for the function of MuSCs. In contrast, fibronectin treatment restores the regenerative capacity of aged MuSCs [149]. Niche-derived nuclear factor B (NF-κB) signaling increases with aging and impairs MuSC function. Consistently, administration of an NF-κB inhibitor restores the lost function of MuSCs [150]. The JAK/STAT pathway is functionally involved in the maintenance of self-renewal capabilities in MuSCs [79,142], and the inhibition of JAK/STAT pathway improves the regenerative capacity of MuSCs [79].

In addition to the stem cell niche, aging also affects circulating signals that directly or indirectly affect the functions of tissue stem cells. These signals include soluble molecules secreted by various tissues, such as hormones, cytokines, growth factors, exosomes, and circulating microRNAs. Wnt ligand levels are higher in serum from aging mice, and canonical Wnt signaling directly antagonizes Notch signaling in MuSCs. The level of TGF-β is significantly increased in serum from older humans and mice, and this contributes to damage and senescence of MuSCs. However, blockage of TGF-β signaling can reverse the regenerative activity of MuSCs in aged mice [151]. The treatment of aged mice with growth differentiation factor 11 (GDF11) or oxytocin reverses the dysfunction of aged MuSCs and restores regenerative function [144,152].The cell surface receptors for β1-integrin and fibronectin are dysregulated in aged MuSCs, and reconstitution of these components can restore the muscle regenerative capacity [153]. Thus, the antibody of β1-integrin (anti-Integrin beta 1/CD29 (TS2/16)) is one of the targets for rejuvenating aged MuSCs. The granulocyte colony-stimulating factor (G-CSF) receptor (G-CSFR) is expressed in developing myoblasts in mouse embryos during the mid-gestation stage [106], and G-CSFR is asymmetrically expressed in activated satellite cells. G-CSF promotes long-term regenerative potential in DMD model mice [117]. Pharmacological interventions to promote MuSC self-renewal are of therapeutic interest to extend the limits of muscle repair with age [154]. Table 1 summarizes the non-cell autonomous rejuvenation strategies to rejuvenate aged MuSCs [123].

### 4.3. Regenerative Strategy

Cell transplantation therapy is one of the strategies to fulfill the pool of healthy MuSCs [154]. The cell-based therapy is based on the transplantation of autologous or heterologous MuSCs with the goal of regenerating the damaged tissue [155]. Myoblasts can be harvested from the muscle biopsy samples and can be expanded in vitro. Thereby myoblasts were main cell sources for cell transplantation therapy. Myoblast transplantations were investigated in animal models and human. To establish the effective regenerative therapy, there are still several problems to be overcome, such as inefficient engraftment, cell death, immune rejection, and requirement of repetitive injections [154,156].

For therapy of several types of muscular dystrophies, human induced pluripotent stem cells (hiPSCs) have great promise for cell sources for regenerative therapy. hiPSCs can differentiate into any type of cells including myoblasts and mature skeletal muscle cells [157,158]. hiPSCs can be generated from the patient’s somatic cells, and transplantation of hiPSC-derived cells do not induce immune rejection in the patient as in heterologous transplantation [155]. Furthermore, gene mutation can be corrected by genome editing technology in patient-derived iPSCs [159], and gene-corrected iPSC-derived cells may be ideal cell sources for regenerative therapy. In DMD, innovation is still necessary to develop the method to deliver the cells to whole body.

## 5. Conclusions

In the adult stage, skeletal muscles have their own stem cells, which can regenerate injured muscles. However, their regenerative potential is impaired during physiological and pathological aging. To develop a therapeutic approach for skeletal muscle regeneration, it is important to understand the molecular mechanisms in how stem cells are maintained and contribute to the regeneration. Adult MuSCs carry self-renewal and multipotent differentiation abilities while being a heterogeneous population. At the individual cell level, the nature of MuSCs differ in their self-renewal, proliferation, and myogenic differentiation potential. These different characteristics of MuSCs contribute to the variety of physiological necessities, such as (1) maintenance of a sustainable reservoir of MuSCs, (2) rapid production of a sufficient number of myogenic progenitor cells, and (3) generation of functional mature contractile muscle cells. Heterogeneous MuSCs show a hierarchical pattern, including genuine stem cells and their progenies. This hierarchical pattern is altered during regeneration and is recovered after regeneration. The nature of MuSCs is affected by many factors such as cellular stress, inflammation, cellular interaction, repetitive regeneration, and the surrounding extracellular matrix.

Restorative interventions to these factors hold promise for possible therapies in regenerative medicine for many age-related diseases and dysfunctions. Among these diseases, DMD and other types of muscular dystrophies are especially important to study considering their clinical severity. The prevalence of such diseases is relatively high and there are currently no fundamental therapies available. The dysfunction in MuSCs is manifested in aged individuals. One of the pathologic mechanisms considered in DMD and other types of muscular dystrophy is stem cell aging due to repetitive injury and regeneration. Further studies should focus on translating the successful rejuvenating regimes of basic medicine into clinical therapies of aged-associated diseases.

## Figures and Tables

**Table 1:** Non-cell autonomous rejuvenation strategies of aged MuSCs.

Intrinsic Rejuvenating Strategies of Aged MuSC			
Target	Mechanism	Function	Reference
Fgfr1 inhibitor SU5402 Spry1 overexpression	reducing FGF signaling	loss of quiescence, regenerative capacity	[81]
Fibronection injection	rescue FAK signaling	proliferative and myogenic potential	[149]
Sodium salicylate	inhibition of NF-κB signaling	regenerative capacity	[150]
TS2/16	activation of b1-integrin/FGFR	regenerative capacity	[153]
Tyr AG 490	inhibition of JAK/STAT	MuSC number; self-renewal; regenerative capacity	[79]
5,15 diphenylporphrine	inhibition of JAK/STAT	MuSC number; self-renewal; regenerative capacity	[79]
**Systemic environment to reverse aging of MuSC**			
**Target**	**Mechanism**	**Rejuvenation on Function**	**Reference**
Frizzled-related protein 3 (sFRP3) incubation	suppression of Wnt signaling	proliferative potential; muscle regeneration	[76]
Dickkopf-1 (Dkk1) injection	suppression of Wnt signaling	muscle regeneration	[76]
TGF-beta receptor kinase inhibitor	attenuating TGFb signaling	regenerative potential	[151]
Growth differentiation factor 11 (GDF11) injection	unknown	regenerative potential	[144]
Oxytocin	activation of MAPK/ERK signaling	MuSC activation and proliferation; regenerative potential	[152]
